# *GmTCP* and *GmNLP* Underlying Nodulation Character in Soybean Depending on Nitrogen

**DOI:** 10.3390/ijms24097750

**Published:** 2023-04-24

**Authors:** Yunchol Kim, Jinhui Wang, Chao Ma, Cholnam Jong, Myongil Jin, Jinmyong Cha, Jing Wang, Yang Peng, Hejia Ni, Haibo Li, Mingliang Yang, Qingshan Chen, Dawei Xin

**Affiliations:** College of Agriculture, Northeast Agricultural University, Harbin 150036, China; 13134518306@163.com (Y.K.); jinhuiwang113@126.com (J.W.); mcneau@163.com (C.M.); 13030046075@163.com (C.J.); j15504506210@163.com (M.J.); chejinming1983@163.com (J.C.); wangjing20211029@163.com (J.W.); b210301014@neau.edu.cn (Y.P.); nhjwinner@163.com (H.N.); lhb2172637105@163.com (H.L.); yml5419@126.com (M.Y.)

**Keywords:** TCP transcription factor, nodulation, nitrate concentration

## Abstract

Soybean is a cereal crop with high protein and oil content which serves as the main source of plant-based protein and oil for human consumption. The symbiotic relationship between legumes and rhizobia contributes significantly to soybean yield and quality, but the underlying molecular mechanisms remain poorly understood, hindering efforts to improve soybean productivity. In this study, we conducted a transcriptome analysis and identified 22 differentially expressed genes (DEGs) from nodule-related quantitative trait loci (QTL) located in chromosomes 12 and 19. Subsequently, we performed functional characterisation and haplotype analysis to identify key candidate genes among the 22 DEGs that are responsive to nitrate. Our findings identified *GmTCP* (TEOSINTE-BRANCHED1/CYCLOIDEA/PCF) and *GmNLP* (NIN-LIKE PROTEIN) as the key candidate genes that regulate the soybean nodule phenotype in response to nitrogen concentration. We conducted homologous gene mutant analysis in *Arabidopsis thaliana*, which revealed that the homologous genes of *GmTCP* and *GmNLP* play a vital role in regulating root development in response to nitrogen concentration. We further performed overexpression and gene knockout of *GmTCP* and *GmNLP* through hairy root transformation in soybeans and analysed the effects of *GmTCP* and *GmNLP* on nodulation under different nitrogen concentrations using transgenic lines. Overexpressing *GmTCP* and *GmNLP* resulted in significant differences in soybean hairy root nodulation phenotypes, such as nodule number (NN) and nodule dry weight (NDW), under varying nitrate conditions. Our results demonstrate that *GmTCP* and *GmNLP* are involved in regulating soybean nodulation in response to nitrogen concentration, providing new insights into the mechanism of soybean symbiosis establishment underlying different nitrogen concentrations.

## 1. Introduction

Soybean is considered one of the most important crops due to the high oil and protein content in its seeds [1]. As the demand for soybeans continues to increase in many countries around the world, scientific cultivation and management of soybean farming have become essential issues. Legumes have a unique feature that distinguishes them from other plants: symbiosis with *Rhizobium*. In other words, legumes have the ability to fix nitrogen for root nodule formation through symbiosis with *Rhizobium* [2]. Biological nitrogen fixation is a process in which nitrogen is converted into a usable form with the help of living organisms. Soybean has the capacity to form symbiosis with *Rhizobium*, which allows it to acquire nitrogen from the air [3,4].

Identifying the genes responsible for establishing symbiotic interactions between soybean and *rhizobium* is essential for understanding the molecular mechanism underlying their interaction. In recent years, the development of molecular marker analysis techniques and the construction of molecular genetic maps, quantitative trait-related QTL analysis and gene cloning of many crops have become a hot spot in molecular breeding [5,6]. The in-depth analysis of quantitative genetic traits is closely related to the development of modern molecular biology techniques [7]. The application and development of molecular markers, population mapping and statistical analysis methods have greatly improved the efficiency of candidate gene discovery [8,9]. In one study, a total of 22 QTLs for the two nodule traits (nodule number and nodule dry weight) were mapped to 12 different chromosomes in the soybean genome, and 17 candidate genes were selected by the previous RNA-seq analysis after infection with *Sinorhizobium fredii* HH103 (a fast-growing rhizobial strain) [10]. *S. fredii* strain HH103 was isolated from a soil sample of Honghu County (Hubei, China) and was first described in 1985, and through its complete genome sequencing and annotation provided a valuable basis for the study of *S. fredii* HH103 and soybean [11].

The TEOSINTE-BRANCHED1/CYCLOIDEA/PCF (TCP) transcription factors are a family of plant-specific proteins that play critical roles in plant growth, development and stress response [12,13]. TCP transcription factors are characterised by a conserved region, a non-canonical basic helix-loop-helix (bHLH) DNA-binding domain called the TCP domain [14,15]. TCP proteins can be divided into two classes. Class I lacks four conservative amino acids in the structure, whereas class II has four conservative amino acids inserted. Class II can be further subdivided into CIN and CYC based on sequence differences [16,17]. In recent years, the regulatory roles of various TCP transcription factors have been extensively studied [18,19]. *Arabidopsis TCP14* and *TCP15* can regulate plant height [20], while *TCP11* and *TCP16* can regulate the vascular bundle and pollen development [21,22]. In addition, *TCP3* can regulate leaf development [23], and *TCP17* can regulate hypocotyl elongation by regulating light and auxin signalling [24]. *Arabidopsis TCP20* is involved in the regulation of shoot, flower and embryo development. In particular, *TCP20* has a tubular function in the systemic signalling pathway of nitrate uptake by the *Arabidopsis* root system [25,26]. *TCP20* mutants have normal primary and lateral root growth in uniform nitrate media, but lateral root growth is inhibited in non-uniform media [17]. In addition, even when ammonium ions are uniformly present in the culture medium, the preferential lateral root growth of the mutant is still clearly inhibited. In the *TCP20* mutant complemented by the transformation of the *TCP20* cDNA clone, the preferential lateral root growth was restored to WT levels [17]. These data demonstrate that *TCP20* regulates nitrate responses and root development. A total of 54 GmTCPs have been identified in soybean, and the GmTCPs have been divided into two homology classes: Class I and Class II. Class II was further subdivided into two subclasses: CIN and CYC/TB1 [27,28]. *GmTCP1*, *GmTCP2*, *GmTCP6*, *GmTCP8*, *GmTCP12*, *GmTCP17* and *GmTCP43* were the best orthologues of *Arabidopsis AtTCP14* and *AtTCP15*, suggesting their potential functions in plant height regulation [27]. One researcher found that overexpression of the flowering repressor gene *E1* could affect leaf development in soybean by directly repressing a large number of leaf development-related CIN-type TCP genes, thus regulating leaf development and flowering time [29]. *GmTCP30* and *GmTCP33* respond to drought stress, *GmTCP42* to heat stress and *GmTCP47* to salt and heat stress [27]. In a study, *Agrobacterium*-mediated transformation was used to introduce the CRISPR/Cas9 expression vector into the soybean cultivar ‘Williams 82’ and generate targeted mutants of the *GmTCP19L* gene, which has been implicated in soybean responses to *Phytophthora sojae* [30]. Although the TCP gene has recently been found to play an important role in abiotic stress and hormone signalling responses in many plant species, it is still unexplored how TCP family genes affect growth and responses to abiotic stress, and whether TCP genes involved in nitrate response affect nodulation or not.

NIN-LIKE PROTEIN (NLP) consists of three domains including the N-terminal conserved domain, an RWP-RK DNA-binding domain and a PB1 domain. The N-terminal conserved domain is required for the nitrate response, while the RWP-RK domain is essential for protein–protein interaction [31]. The PB1 domains adopt a ubiquitin-like β-grasp fold containing two α-helices and a mixed five-stranded β-sheet, and are classified into groups containing an acidic OPCA motif (type I), the invariant lysine residue on the first β-strand (type II), or both (type I/II). The PB1 domain is also a conserved domain required for protein–protein interaction [32]. Although the PB1 domain of NLP transcription factors appears to mediate protein–protein interactions associated with nitrate-inducible gene expression in higher plants, its precise role in nitrate-inducible gene expression has not been characterised [33]. In the model legume *Lotus japonicus*, LjNLP1—previously identified as a NIN-like protein—directly regulates the expression of *LjNRT2.1* and *LjNLP4*, which are involved in regulating the expression of nodulation-related genes [34,35]. In *Medicago truncatula*, *MtNLP1* also inhibits nodulation by directly inducing a negative regulator of nodulation, *MtCLE35* [34]. *NLP6* and *NLP7* are activators of nitrate assimilation genes [26]. *AtNLP6* and *AtNLP7* have been identified as key transcription factors for the primary nitrate response (PNR) in *Arabidopsis* [33]. Under N starvation, TCP20-NLP6&7 heterodimers accumulate in the nucleus and then down-regulate the expression of the G2/M cell cycle marker gene *CYCB1;1* and up-regulate nitrate assimilation [26]. The NLP protein is a homologue of NIN (NODULE INCEPTION) and is found in a variety of plants. *GmNINa* interacted with *NNC1* (Nodule Number Control 1) and predominantly inhibited the transcriptional activation of *GmRIC1* (Rhizobia-Induced CLE1), *GmRIC2* and *miR172c* by *GmNINa*, thereby controlling nodulation [36]. The NIN-like protein (NLP) transcription factors *NLP2* and Nodule Inception (NIN) directly activate the expression of leghemoglobins through a promoter motif that resembles a ‘double’ version of the nitrate responsive elements (NREs) targeted by other NLPs, and has conserved orientation and position across legumes [37]. Few studies have been carried out on NLP in soybean. TCP and NLP proteins belong to an evolutionarily conserved family that appears to play ancient roles. TCP and NLP proteins may be an important factor in the nitrate response. In soybean, TCP and NLP are involved in the nitrate response pathway and affect symbiotic nitrogen fixation.

There is a significant interaction between soil nitrogen content and symbiosis establishment. In this study, genes’ responses to *rhizobium* infection were identified by mining the different expression genes induced by *rhizobium*. Two candidate genes, *GmTCP* and *GmNLP*, were identified. Complementation analysis of *GmTCP* and *GmNLP* in *Arabidopsis* response to nitrogen content was performed. Moreover, the roles of *GmTCP* and *GmNLP* regulating the nodule phenotype depending on different nitrogen contents were elucidated. Our results will contribute to the functional analyses of *GmTCP* and *GmNLP* genes and genetic modification in the soybean symbiotic character.

## 2. Results

### 2.1. Identification of Candidate Genes Associated with Nodulation Traits

**Figure 1:**
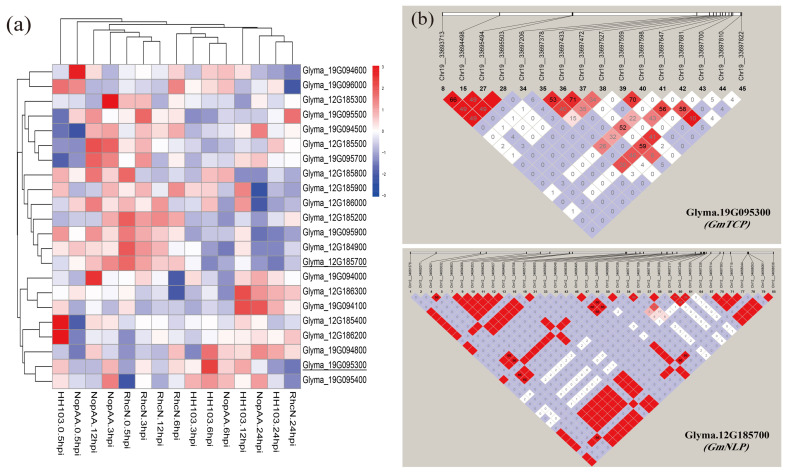
The expression profiles of DEGs and haplotype analysis. (**a**) Heat map of DEGs located in QTL locus. (**b**) Haplotype analysis of two candidate genes in the chromosome segments’ substituted lines.

In the previous RNA-seq analysis, we determined two chromosome regions that might respond to rhizobium induction and identified 22 DEGs in those two regions (Figure 1a). Among the DEGs, *Glyma.19G095300*, *Glyma.19G095400* and *Glyma.19G094800* exhibited a tendency for their expression levels to gradually increase until 6 h of infection by *Sinorhizobium fredii* HH103. Conversely, the expression levels of *Glyma.12G185700*, *Glyma.12G184900*, *Glyma.19G095900*, *Glyma.12G185200* and *Glyma.12G186000* decreased after infection with *S*. *fredii* HH103. Furthermore, we also detected the single nucleotide polymorphism support of *GmTCP* and *GmNLP* in the chromosome segments’ substituted lines [10]. A high degree of single nucleotide polymorphism (SNP) between the parents and lines of chromosome segments’ substituted lines was identified [10] (Figure 1b). These results led us to the hypotheses that *GmTCP* and *GmNLP* could respond to rhizobium.

### 2.2. TCP and NLP Homolog Genes’ Responses to the Nitrogen Concentration in Arabidopsis

**Figure 2:**
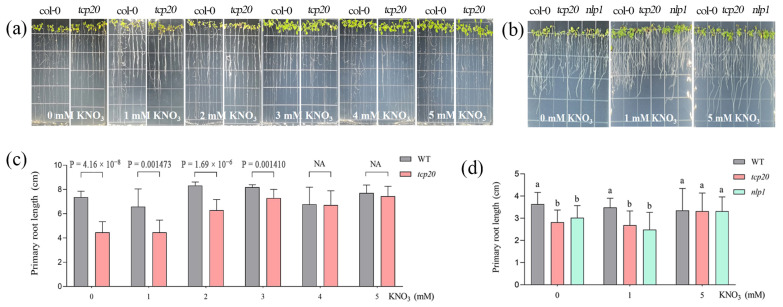
Root phenotype and primary root length of WT, single mutants under 0~5 mM nitrate treatment. (**a**) Root phenotype of WT, *tcp20* mutants under 0~5 mM nitrate treatment. (**b**) Root phenotype of WT, *nlp1* mutants under 0, 1, and 5 mM nitrate treatment. (**c**) Primary root length of WT, *tcp20* mutants under 0~5 mM nitrate treatment. (**d**) Primary root length of WT, *nlp1* mutants under 0, 1, and 5 mM nitrate treatment. For root phenotype and primary root length, *Arabidopsis* seedlings were grown on 5 mM KNO_3_ plates for 6 days and then were transferred to new 0~5 mM KNO_3_ plates for three days. Error bars show SEM (*n* = 10). One-way ANOVA was performed and followed by *t*-test to calculate the *p*-value (using WT as control). Different letters represent statistic difference between WT and mutants under same nitrate condition (*p* < 0.05).

Firstly, in order to study the function of candidate genes in *Arabidopsis thaliana*, sequence blast was used to identify the homolog protein with *GmTCP/GmNLP* in *Arabidopsis thaliana.* The homology between these genes and *GmTCP/GmNLP* was found to be 61.0% and 86.5%, respectively. Since the TCP and NLP family are related to nitrogen response, it is important to understand their function. We detected the gene response to different nitrogen levels in *Arabidopsis thaliana*. Using the knockout mutant, we could also detect the complementation effect of *GmTCP/GmNLP* on different nitrogen contents. Furthermore, we investigated how the primary root growth of the *tcp20* mutant responded to nitrate concentration. Specifically, the change in the primary root length phenotype of the *tcp20* mutant was examined across nitrate concentrations ranging from 0 to 5 mM. Under nitrate starvation, the primary root length of the *TCP20* mutant was significantly different from that of the wild type. However, at high nitrate concentrations (5 mM KNO_3_), the difference in primary root length between the mutant and the wild types was not apparent. At lower nitrate concentrations, the primary root length of the *TCP20* mutant was significantly shorter, but this difference gradually decreased at higher concentrations (Figure 2a,c).

It has recently been reported that the NIN-like protein LjNLP1 directly regulates the expression of *LjNRT2.1* and *LjNLP4*, as well as nodulation-related genes. It has also been shown that certain NLP genes encode the factors that activate the nitrate assimilation genes. To further investigate the response of *NLP1* to nitrate concentrations, primary root length was examined in the nlp1 mutant. The results showed a significantly reduced primary root growth under N starvation and low nitrate concentrations; however, this difference was not significant under high nitrate conditions (Figure 2b,d). Multiple comparison analyses showed that there was no correlation between *AtTCP20* and *AtNLP1*. Two-way ANOVA analysis showed that the interaction effect between N treatment and genotypes was statistically significant in regulating growth phenotypes, such as primary root length (*p* < 0.05).

Finally, the mutation of these genes resulted in a limitation of the primary root length under N starvation and low nitrate concentrations, suggesting that they may encode key factors in the development of nitrate-responsive root formation.

### 2.3. Response of GmTCP and GmNLP to Different Nitrate Concentrations

**Figure 3:**
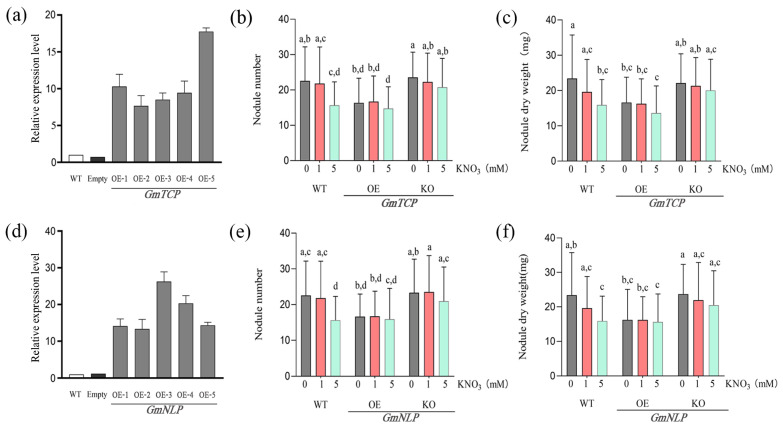
WT, overexpression, and mutant lines’ response to different nitrate concentrations in soybean. (**a**,**d**) The identification of *GmTCP* and *GmNLP* overexpression. (**b**,**c**) Overexpression, knockout of *GmTCP* response to different nitrate concentrations. (**e**,**f**) Overexpression, knockout of *GmNLP* response to different nitrate concentrations. The nodule phenotype (nodule number, nodule dry weight) of WT, overexpression and mutant lines was observed 28 days after inoculation with *S*. *fredii* HH103. Error bars show SEM (*n* = 20). Different letters indicate statistically significant differences (*p* < 0.05, one-way ANOVA followed by multiple comparisons).

Previous experiments on *AtTCP20* and *AtNLP1* confirmed that these genes are involved in the nitrate response signalling pathway. We further investigated the functions of *GmTCP* and *GmNLP* in the soybean nitrate response pathway using *GmTCP/GmNLP* transgenic hairy roots. Six plants were selected from each overexpressed line and used for RNA extraction and the subsequent evaluation of *GmTCP/GmNLP* expression by qRT-PCR. The expression of *GmTCP* and *GmNLP* was significantly higher than that of the empty vector (Figure 3a,d). Three lines with the highest *GmTCP/GmNLP* expression levels were then used for further phenotype analysis. In the knockout transgenic lines, a mutation effect of two to seven base deletions in a target region was observed, and the nodulation phenotype of plants with this mutation was observed at different nitrogen concentrations. The overexpression of *GmTCP* had a negative effect on nodule number and nodule dry weight (Figure 3b), although the nodule number was reduced more at high nitrogen concentrations than at low nitrogen concentrations (0 mM and 1 mM KNO_3_) (Figure 3b). When *GmTCP* was knocked out, the nodule number and nodule dry weight had no significant difference but at the high nitrogen concentration (5mM KNO_3_) (Figure 3b,c). These results support *GmTCP* being able to regulate the nodule phenotype depending on the high nitrogen concentration.

The overexpression of *GmNLP* also showed a slight difference with increasing nitrate concentrations, but it was significantly reduced compared to the wild type. The *GmNLP* mutant lines showed some reduction in nodule formation with increasing nitrate concentrations (Figure 3e,f). Under different nitrogen concentrations, the difference in nodule phenotype was not significant, however, compared with the wild type at high nitrogen concentration being able to inhibit the nodule number, and the knockout mutant of *GmNLP* could recover the nodule phenotype as wild type at a lower nitrogen concentration (Figure 3e,f). Taken together, these results support *GmTCP* and *GmNLP* being involved in the formation and regulation of nodulation at a high nitrate concentration but not at a low nitrogen concentration.

### 2.4. Effects of GmTCP and GmNLP on the Nodulation

**Figure 4:**
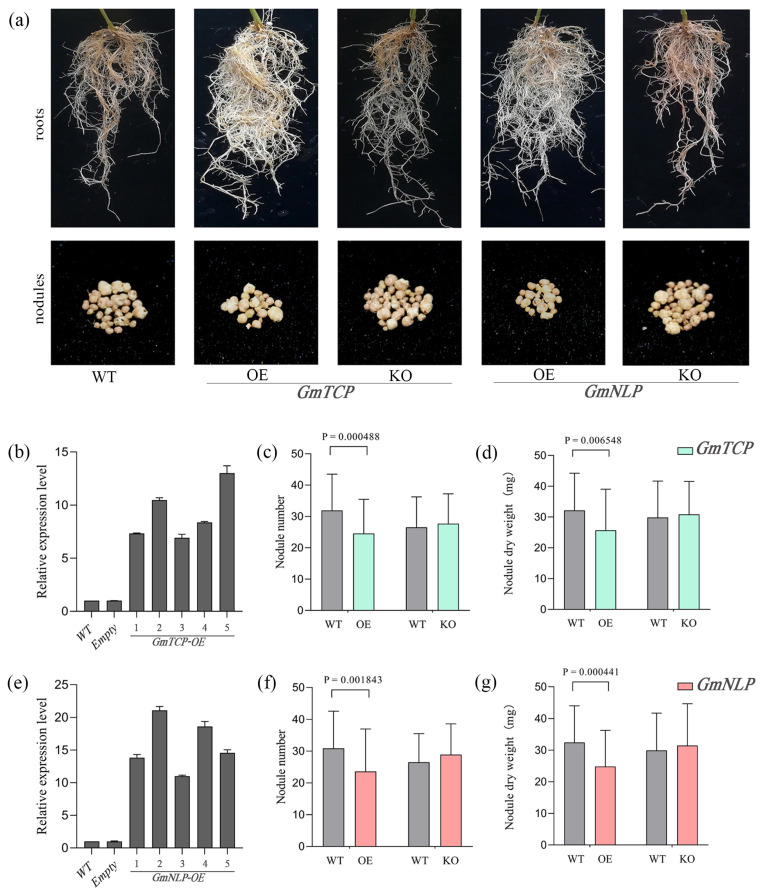
The influence of WT, overexpression and knockout of *GmTCP* and *GmNLP* in nodule. The growth of transgenic hairy roots 30 d after inoculation with *S*. *fredii* HH103. KO showed that the target gene was silenced in hairy roots, OE showed that the target gene was overexpressed in hairy roots, WT, and soybean hairy roots with the empty vector pSOY1-hairyred. (**a**) Photographs of root development and growth performance of nodules. (**b**) The identification of overexpression of *GmTCP*. (**c**) The nodule number of overexpression, knockout of *GmTCP*. (**d**) The nodule dry weight of overexpression, knockout of *GmTCP*. (**e**) The identification of overexpression of *GmNLP*, (**f**) the nodule number of overexpression, knockout of *GmNLP*. (**g**) The nodule dry weight of overexpression, knockout of *GmNLP*. The nodule phenotype (nodule number, nodule dry weight) of WT, overexpression and mutant were observed 28 days after inoculation with *S*. *fredii* HH103. Error bars show SEM (*n* = 20). Different letters indicate statistically significant differences (*p <* 0.05, one-way ANOVA followed by multiple comparisons).

We demonstrated that *GmTCP* and *GmNLP* were involved in the formation and regulation of nitrate-induced nodulation. To investigate the effects of *GmTCP* and *GmNLP* on nodule phenotype, overexpression and knockout lines were constructed via the soybean hair root system. The effects of *GmTCP* and *GmNLP* on the nodule number and nodule dry weight were detected at 28 post inoculation with *S*. *fredii* HH103. The five plants were selected from the overexpressed line and used for RNA extraction and subsequent evaluation of the *GmTCP*/*GmNLP* expression through qRT-PCR. The expression was significantly higher in *GmTCP* and *GmNLP* than in the empty vector (Figure 4b,e). These lines were used for further phenotype analysis. In knockout transgenic lines, a mutant effect of two to seven base deletions was observed and the nodulation phenotype of plants with this mutation was observed at different concentrations. It was observed that when compared with the wild type, the overexpression of *GmTCP* resulted in a decrease in the nodule number and the nodule dry weight compared to the wild type. This finding was consistent with the above experimental data that showed the inhibition of the nodulation in the overexpression lines. In contrast, the mutant had a nodule phenotype that was almost indistinguishable from or slightly higher than that of the wild type. This indicates that *GmTCP* function loss resulted in the removal of the inhibition of nodulation (Figure 4a,c,d). Consistent results were obtained for *GmNLP*; the overexpression resulted in a more reduced nodule phenotype than that of the wild type, while the mutant had a higher nodule phenotype than that of the wild type (Figure 4a,f,g). These results support the upregulation of *GmTCP* and *GmNLP* being able to inhibit nodule formation, and the result of the nodule number and nodule dry weight, suggesting that *GmTCP* and *GmNLP* are involved in the regulation of nodulation.

### 2.5. Expression Pattern of GmTCP during Root Growth and Nodulation

**Figure 5:**
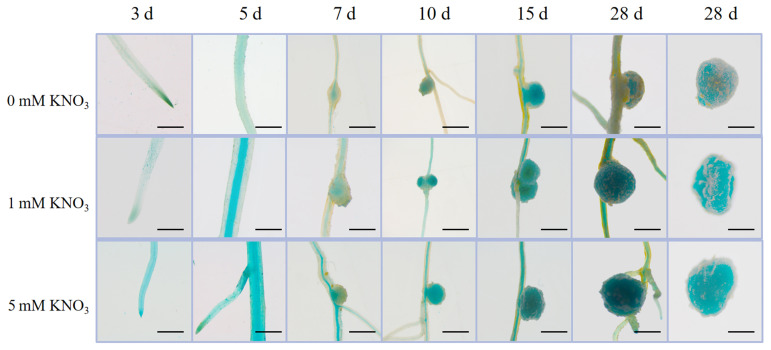
After inoculation with rhizobia, at different nitrate concentration expressions and localisation patterns of *GmTCPpro::GUS* during nodule and lateral root formation.

To determine the spatial expression pattern of *GmTCP*, we performed a promoter-GUS reporter analysis. First, the promoter fragment (3.0 Kb) of *GmTCP* was constructed on the pCAMBIA3301 vector. The *GmTCPpro:GUS* construct showed that *GmTCP* was expressed throughout root development and nodulation. At the stage of nodulation, the *GmTCPpro:GUS* construct showed strong GUS expression throughout the nodule. In addition to its expression in the nodulation cell line, *GmTCP* was also expressed in the roots, especially in the root tips (Figure 5). Furthermore, the higher the nitrate concentration, the higher the GUS expression. These results, that *GmTCP* was expressed in roots and nodules, support that *GmTCP* was involved in the formation and regulation of nodulation under varying nitrate concentration. The promoter activity analysis showed as a primary result that the high nitrogen concentration might have more activity to active the promoter function than lower nitrogen concentration at 5 d post inoculation with *rhizobium*.

## 3. Discussion

In this study, we identified the candidate genes under soybean nodule character response to nitrogen concentration by integrating QTL mapping, RNA-seq data and single nucleotide polymorphism analysis, and we characterised the function of two candidate genes.

*AtTCP20*, the *Arabidopsis* homologous gene of *GmTCP*, has been known to regulate genes related to bud, embryo, and flower development. In particular, *AtTCP20* can bind to 100 or more nitrate-induced genes, being a crucial factor in the nitrate signalling pathway [17,25,26]. In this study, there was found a significant difference in primary root length between the *AtTCP20* mutant and wild type under nitrogen-free conditions, while under high concentrations of nitrate (5 mM KNO_3_), no significant difference in primary root length was observed between the mutant and the wild type. Additionally, at low nitrate concentrations, the primary root length of *AtTCP20* mutants and wild types was significantly different, but the difference decreased as the concentration of nitrate increased. These results demonstrate that *AtTCP20* responds specifically to nitrate starvation or imbalance and then regulates root growth. Previous research on single mutants of *AtTCP20* showed normal primary and lateral root growth on a homogenous nitrate medium, but lateral root growth was restricted on a heterogeneous nitrate medium. *AtTCP20* mutants still inhibited lateral root growth on a uniform ammonium salt medium, suggesting that *AtTCP20* may respond to other nitrogen sources such as ammonium salt [17]. *TCP20* binds to the GCCCR motif in the promoter of the mitotic cyclin gene *CYCB1;1*, regulating its expression. Under nitrogen-free conditions, the mRNA level of *CYCB1;1* was significantly increased in the whole root of *TCP20* mutants, indicating that nitrogen starvation induced the expression of the *TCP20* gene. The mutation of *TCP20* resulted in the improper regulation of *CYCB1;1*, thereby causing a premature exit from the cell cycle and eventually inhibiting root growth [26]. In summary, *AtTCP20* is an important factor that responds to nitrogen starvation or imbalance. We also studied the response of *AtNLP1* mutant plants to nitrate concentration in *Arabidopsis*. Under nitrogen-free and low nitrate conditions, the primary root length of *NLP1* mutants was significantly limited, whereas under high concentrations of nitrate the difference was not significant. *NLP6/7* regulates the expression of *CYCB1;1* in the same way as *TCP20*, ultimately leading to limited primary root development [26]. To date, there have been few reports on the roles of *NLP*, particularly on the molecular mechanism of *NLP* in plant growth and abiotic stress.

The *GmTCP* gene may also respond to nitrogen status. In order to verify the responses of *GmTCP* overexpression lines to different nitrate concentrations, this study constructed *GmTCP* overexpression and knockout transgenic lines via hairy root transformation and examined their nodulation responses after treatment with different concentrations of nitrate and the inoculation of *S. fredii* HH103. The results showed that in hairy roots transformed with empty vector, NN and NDW were significantly reduced under high nitrate concentration. Under low nitrate concentration, compared with nitrogen deficiency, NN and NDW decreased, but the effect was not significant. In *GmTCP* overexpressing hairy roots, NN and NDW decreased with the increase in nitrate concentration. However, compared with the wild type, the nodulation of *GmTCP* overexpressing hairy roots was significantly limited, and NN and NDW were significantly reduced. This experiment shows that *GmTCP* gene overexpression can inhibit nodulation at different nitrate concentrations; in *GmTCP* knockout hairy roots, NN and NDW decreased slightly with the increase in nitrate concentration, but the nodule phenotype of *GmTCP* knockout hairy roots did not differ much from that of hairy roots transformed with the empty vector under nitrogen-starved conditions, and there was no restriction on the nodule phenotype. Overall, the *GmTCP* gene is involved in the response to different nitrate concentrations. There are many studies on how plants absorb and transport nitrate, and the representative proteins in this process are nitrate transport proteins NRT1 and NRT2. Under N starvation, nitrate can induce the expression of nitrate transporter protein GmNRT2 in soybean roots, thereby increasing the absorption and transport of nitrate by roots [34]. Different nitrogen concentrations in the soil will affect the dry weight and number of nodules, and the activity of nitrogenase will decrease when the nitrogen source is abundant. The activity of nitrogenase is highest under nitrogen-free conditions. This indicates that the nitrogen-fixing activity of *rhizobia* is related to the concentration of nitrate. In hairy roots transformed with empty vector, the *GmTCP* gene may respond to high concentrations of nitrate, thereby regulating the expression of *NRT1* and *NRT2* genes and restricting the nodule phenotype. This hypothesis can explain the results obtained from our experiments on *GmTCP* gene overexpression and mutant strains.

This study also constructed *GmNLP* gene overexpression and knockout transgenic roots. The gene’s response to different nitrate concentrations was tested by treating the roots with various concentrations of nitrate and inoculating them with *S. fredii* HH103. The results showed that in *GmNLP*-overexpressing hairy roots, NN and NDW decreased as the nitrate concentration increased. However, compared with the wild type, overexpression significantly limited the nodulation and reduced both NN and NDW. In *GmNLP*-knockout hairy roots, the nodule phenotype slightly decreased with increasing nitrate concentration, but the nodule phenotype of *GmNLP*-knockout hairy roots did not differ significantly from that of empty vector-transformed hairy roots under nitrogen starvation conditions. In summary, the *GmNLP* gene is involved in responding to different nitrate concentrations. In *L. japonicus*, *LjNLP4* and *LjNLP1* play critical roles in the response to nitrate. High nitrate concentrations can induce *LjNLP1*, which, in turn, can induce *LjNRT2.1* expression [35]. The expression of *MtNRT2.1* is impaired due to mutations in the homologous gene *MtNLP1*. Such *NLP1-NRT2.1* regulatory modules may be conserved in many plants [38]. *LjNLP1* may induce the expression of *LjNRT2.1* when nitrate is present in the culture medium. In turn, the expression of *LjNRT2.1* may promote nitrate uptake and transport, ultimately triggering the accumulation of *LjNLP4* in the nucleus. Then, *LjNLP4* may induce or suppress the expression of symbiosis genes to regulate nodule formation [35,39]. This may be a way to respond to nitrate and regulate symbiotic relationships. The tissue-specificity experiment results of *GmTCP* promoter expression showed that the *GmTCP* gene was strongly expressed throughout nodule formation, supporting the hypothesis that the *GmTCP* gene is involved in symbiotic nodule formation. Further experiments are needed to confirm this hypothesis.

In summary, *GmTCP* and *GmNLP* encode important regulatory factors that respond to nitrogen concentrations for soybean nodulation. Further analysis of these genes would be of great significance for better understanding the symbiotic mechanism between soybean and rhizobia, as well as for improving soybean’s nitrogen fixation ability.

## 4. Materials and Methods

### 4.1. Candidate Gene Identification Based on RNA-Seq Data

DEGs (with logFC > 1 and FDR < 0.05) were extracted from the previous RNA-seq data [10] for soybean roots infected with the rhizobium of one wild type and two mutants. Specifically, DEGs were identified in chromosomes 12 and 19, and the chromosome substitution segments were further compared to screen the key root-nodulation-associated candidate genes [7].

RNA-seq was performed on the root samples obtained from the soybean cultivar Suinong 14 (SN14) inoculated with different *rhizobia* at various time points [40]. A total of 180 samples were taken from the roots of SN14 at various points (0.5, 3, 6, 12, and 24 h) after infection with rhizobia injected with HH103, ΩNopAA, or ΩRhcN. For each injection, 3 biological replicates of the plants were used. The TruSeq Stranded mRNA Library Prep Kit from Illumina was used to create PolyA^+^ libraries, and the mRNA sequencing was carried out on the Illumina HiSeq 4000 PE 150 platform [7]. The transcriptome raw sequencing data have been submitted to the NCBI (https://www.ncbi.nlm.nih.gov/bioproject/?term=PRJNA854816, accessed on 15 July 2022) database as individual BioProjects: PRJNA854816.

FastQc and MultiQc were employed to detect the raw Fastq sequence files for the initial quality check. The low-quality reads were trimmed with Trim galore at the criterion of [-q 20] [-length 140] [-e 0.1] [-stringency 3]. Then, FastQc and MultiQc were used again for quality testing to screen qualified reads (19.7 ≤ M SEQS ≤ 32.7 after treatment). Indexes were built by hisat2 with the *Glycine max* genome annotation file Gmax_275_Wm82.a2.v1.gene_exons.gff3 (http://www.phytozome.net/, accessed on 15 July 2022) [41].

### 4.2. Strains, Plant Materials and Growth Conditions

The *E. coli* DH5α cells used were grown at 37 °C in Luria–Bertani (LB) medium [42]. *Sinorhizobium fredii* HH103 were grown at 28 °C in tryptone yeast (TY) medium [43]. All *Arabidopsis thaliana* plants were of Columbia ecotype. The *tcp20* (SALK_041906), *nlp1*(SALK_125809) mutants were obtained from ABRC. The *tcp20* and *nlp1* mutants were produced by selfing the F1 plants and selecting F2 homozygous progeny by PCR analysis.

For primary root length, *Arabidopsis* seedlings were grown on 5mM KNO_3_ plates for 6–7 days and then were transferred to different nitrate concentration plates or N-free plates for three days. The medium used in this experiment was N-free 1/2MS medium.

### 4.3. Plasmid Construct

The CDS fragment of *GmTCP* and *GmNLP* was constructed into the vector Fu28-GFP, and then LR recombination reactions were used to construct the overexpression vector pSOY1-*GmTCP*-GFP and pSOY1-*GmNLP*-GFP (Supplemental Table S1, Figures S1−S10). The online *Crispr-P* tool was used to design the sgRNA primer for these genes, and the designed sqRNA was attached to the pGES201 vectors. The designed sgRNA sequence and the experimental results of the pGES201 vector construction process are shown in Supplemental Table S1, Figures S6–S8.

This fragment, including a 3.0-kb sequence directly upstream of the initiation codon of *GmTCP*, was cloned into pCAMBIA3301-GFP. For promoter-GUS analysis using hairy roots on soybean, a 3.0 kb fragment of the *GmTCP* promoter region was amplified by PCR from WT genomic DNA and cloned upstream of the GUS gene in the pCAMBIA3301-GUS vector (Supplemental Table S1, Figures S9 and S10).

### 4.4. Agrobacterium Rhizogenes-Mediated Transformation of Soybean to Study Root Biology

The sterilised seeds were placed into wet vermiculite at a depth of 1–2 cm which was then transferred into a growth chamber or greenhouse at 28 °C. Meanwhile, *Agrobacterium* strain(s) harbouring the desired construct(s) (from glycerol stock) were inoculated onto the surface of LB plates containing the appropriate antibiotics and incubated at 28 °C for 2 days, then a single colony was transferred onto a fresh plate. The fresh bacterial culture from the plate was suspended in 1 mL of liquid LB medium containing 15% (*v*/*v*) glycerol, and then each 200 μL of the suspension culture liquid was spread onto the surface of four LB plates containing the appropriate antibiotics and incubated at 28 °C overnight. Bacteria from the plates were collected and then inoculated into the healthy plantlets with unfolded green cotyledons by stabbing at the cotyledonary node and/or at the hypocotyl proximal to the cotyledon. The plants were then placed under 12 h light/12 h dark at 28 °C/25 °C in a growth chamber (humid chamber only) for 2–3 weeks until the hairy roots grew to approximately 5–10 cm in length and were long enough to support growth of the plant. During this period, the plants were supplied with sterile B&D solution containing 0~2 mM KNO_3_ as a nitrogen source. When the hairy roots were approximately 5–10 cm in length, the primary root was removed by cutting the hypocotyl ~1 cm under the wounding site where the hairy roots were formed. The six plants were selected from each overexpressed line and used for RNA extraction and subsequent evaluation of *GmTCP*/*GmNLP* expression through qRT-PCR. The highest *GmTCP*/*GmNLP* expression levels were then used for further phenotype analysis. These lines moved into new pots after 5 days’ infection with *S*. *fredii* HH103. After 28 days, the two phenotypes of the nodules in all soybean materials were investigated [44].

### 4.5. Biological Nitrogen Fixation Traits

At 28 days of *S*. *fredii* HH103 inoculation, the two phenotypes of the nodules in all soybean materials were investigated. The NN and NDW were evaluated. To determine the NDW, the nodules were placed in an oven at 65 °C for 36 hr.

Root samples were harvested at 28 days post-inoculation with *S*. *fredii* HH103, and wild-type plants were used as the control. In overexpression lines, the total RNA was isolated from the roots using the TRIzol Reagent (Invitrogen, Carlsbad, CA, USA), and then each RNA sample was converted into cDNA using the HiScript^®^II Q RT SuperMix (Vazyme Biotech Co., Nanjing, China). The qRT-PCR was performed with the TB Green Fast qPCR Mix (TaKaRa, Dalian, China) on the Roche LightCycler 480 II System (Roche, Basel, Switzerland). The qRT-PCR program was as follows: denaturation at 95 °C for 30 s, followed by 40 cycles of 95 °C for 5 s, 58 °C for 20 s and 72 °C for 30 s. All RNA extractions were performed in three biological replicates, and each cDNA sample was analysed three times. Specific primers were designed to the Williams 82 gene sequences retrieved from the Phytozome website [45]. *GmActin* was used as the reference gene to calibrate the transcript abundance values among different samples. Ct values were calculated by the Roche LightCycler 480 II software. In knockout transgenic lines, genome DNA was extracted from the root, the corresponding gene region was amplified with a specific primer, and then sequencing was conducted to see if there was a mutation in the region, and the NN and NDM were measured for the mutated roots.

### 4.6. GUS Staining

Root tissues were gently pre-fixed in 90% acetone (pre-cooled at −20 °C) under vacuum infiltration for 5 min, and then placed on ice for 20 min. Next, they were rinsed with cold water for 5 min and vacuum infiltrated for 5 min on ice with a pre-cooled staining solution. After that, they were incubated at 37 °C for 12 h. Samples were changed through 30 min steps of 20% ethanol, 30% ethanol, 50% ethanol, and FAA. The FAA was then removed, and 70% ethanol was added and the solution was left for two h, during which ethanol was replaced with fresh twice. Finally, the samples were stored at 4 °C before observation with a dissecting microscope [46].

## 5. Conclusions

In this study, we identified two candidate genes, *GmTCP* and *GmNLP*, that regulate soybean nodule phenotype in response to nitrogen. Homologous genes of these two candidates have been shown to be important factors in regulating root development in response to nitrogen concentration in *Arabidopsis thaliana*. Overexpression and gene knockout in *GmTCP* and *GmNLP* in soybean resulted in different responses to different nitrate concentrations, leading to different nodule phenotypes. These results highlight the involvement of *GmTCP* and *GmNLP* in the regulation of soybean nodulation under nitrogen concentration. The role of these genes in the symbiotic mechanism between soybean and rhizobia is of great importance for improving nitrogen fixation efficiency, and therefore further analysis of their functions may provide valuable insights into the mechanism of nodule formation in response to nitrate.

## Data Availability

Not applicable.

## Supplementary Materials

The supporting information can be downloaded at: https://www.mdpi.com/article/10.3390/ijms24097750/s1.
