# Supplementary material for: GmTCP and GmNLP Underlying Nodulation Character in Soybean Depending on Nitrogen
Source: Int J Mol Sci. 2023 Apr 24;24(9):7750. doi: 10.3390/ijms24097750 (PMC10178161; doi:10.3390/ijms24097750)
Supplement: Supplementary file 1 [file ijms-24-07750-s001.zip › ijms-2313501-supplementary.pdf]

Table S1 Primers for this research

CRISPR-TCP-F	GGATTGTGCTGTTGATGGATCCCAA	pSOY1-TCP-F	ATGGATCCCAAGGGCTCAAAGCA
CRISPR-TCP-R	AAACTTGGGATCCATCAACAGCACA	pSOY1-TCP-R	CTACTGTCCTCCTGAGCCTTGAGAATCA
CRISPR-NLP-F	GGATTGCTAATATCGCCACCGCCGAG	pSOY1-NLP-F	ATGGAGAAGGGTAGAACCGTGGTGG
CRISPR-NLP-R	AAACCTCGGCGGTGGCGATATTAGCA	pSOY1-NLP-R	TCATTGGACGGGATTGTTGCCAT
Crispr-F	ggcgggaaacgacaatctgatc	TCP-pro-F	gtacccggggatcctctagaCCAAGTCTCGAAAAGCGTCCA
qRT-TCP-F	CTGAGATCGGTTTGTTTTGTGA	TCP-pro-R	taccctcagatctaccatggCAACAGCACACAAAAGAAAAGAGG
qRT-TCP-R	TAACTTGCAAGTGATGTTCCAC	pCAMBIA F	CCATGGTAGATCTGAGGGTAAATTT
qRT-NLP-F	CGAGGCAACAAGGAAACAATAA	pCAMBIA R	TCTAGAGGATCCCCGGGTACC
qRT-NLP-R	TGAATCAGAATGATGTTCGCAC	qRT-UNK1-R	GGTGGAAGGAACTGCTAACAAT
qRT-UNK1-F	TGGTGCTGCCGCTATTTACTG		

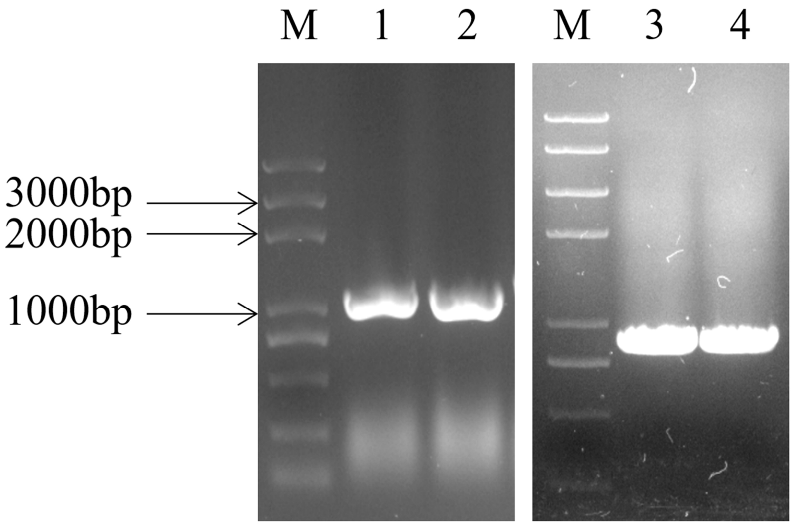

Figure S1. PCR analysis of *pSOY1-Glyma.19G095300, pSOY1-Glyma.12G185700* recombinant vector for plant expression.

Note: M: Trans 2K Plus DNA marker; 1, 2: *Glyma.19G095300* (pSOY1-TCP-F/R); 3, 4: *Glyma.12G185700* (pSOY1-NLP-F/R)

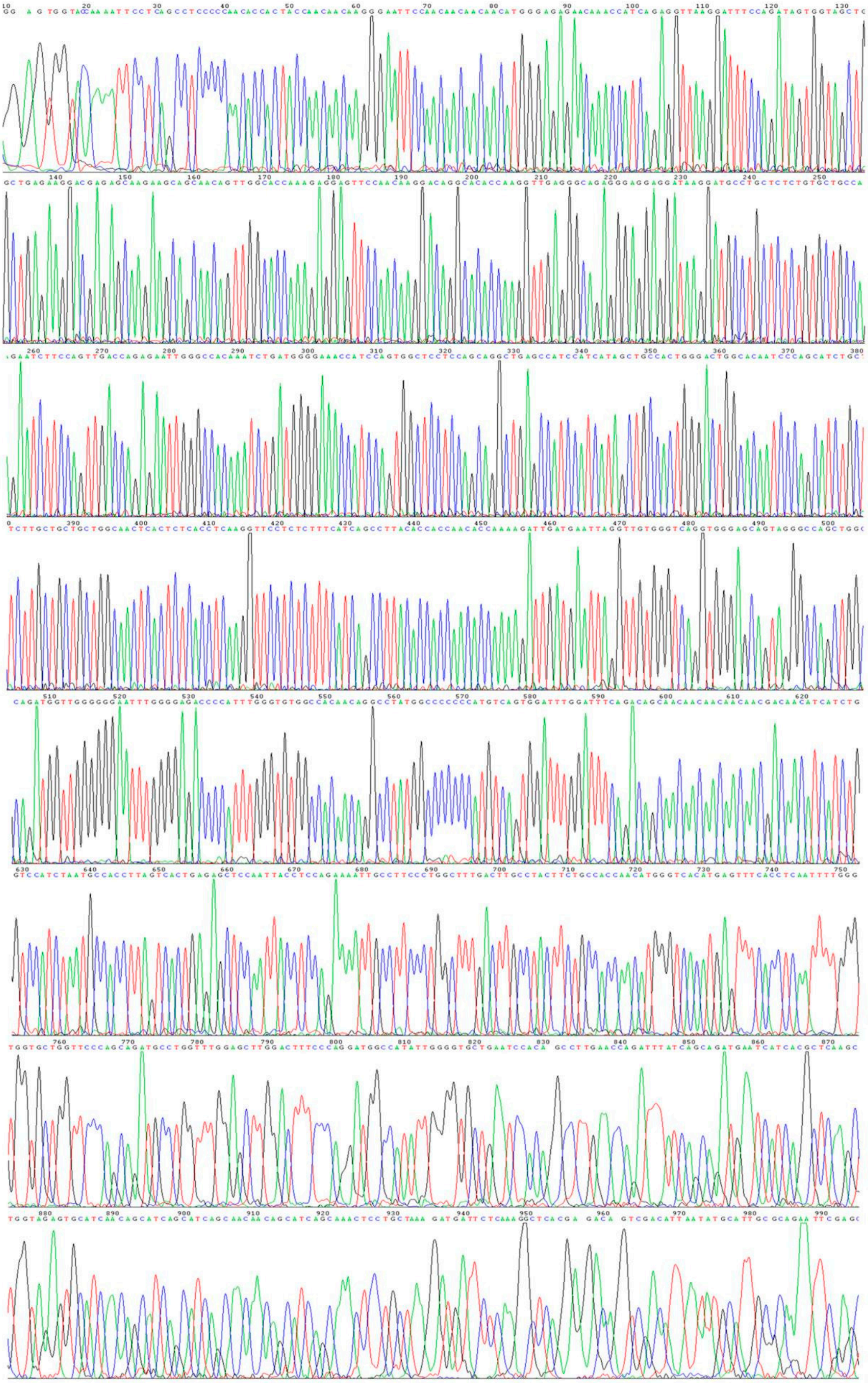

Figure S2. Sequencing of target gene from *pSOY1-Glyma.19G095300* recombinant vector for plant

expression with forward-specific primer.

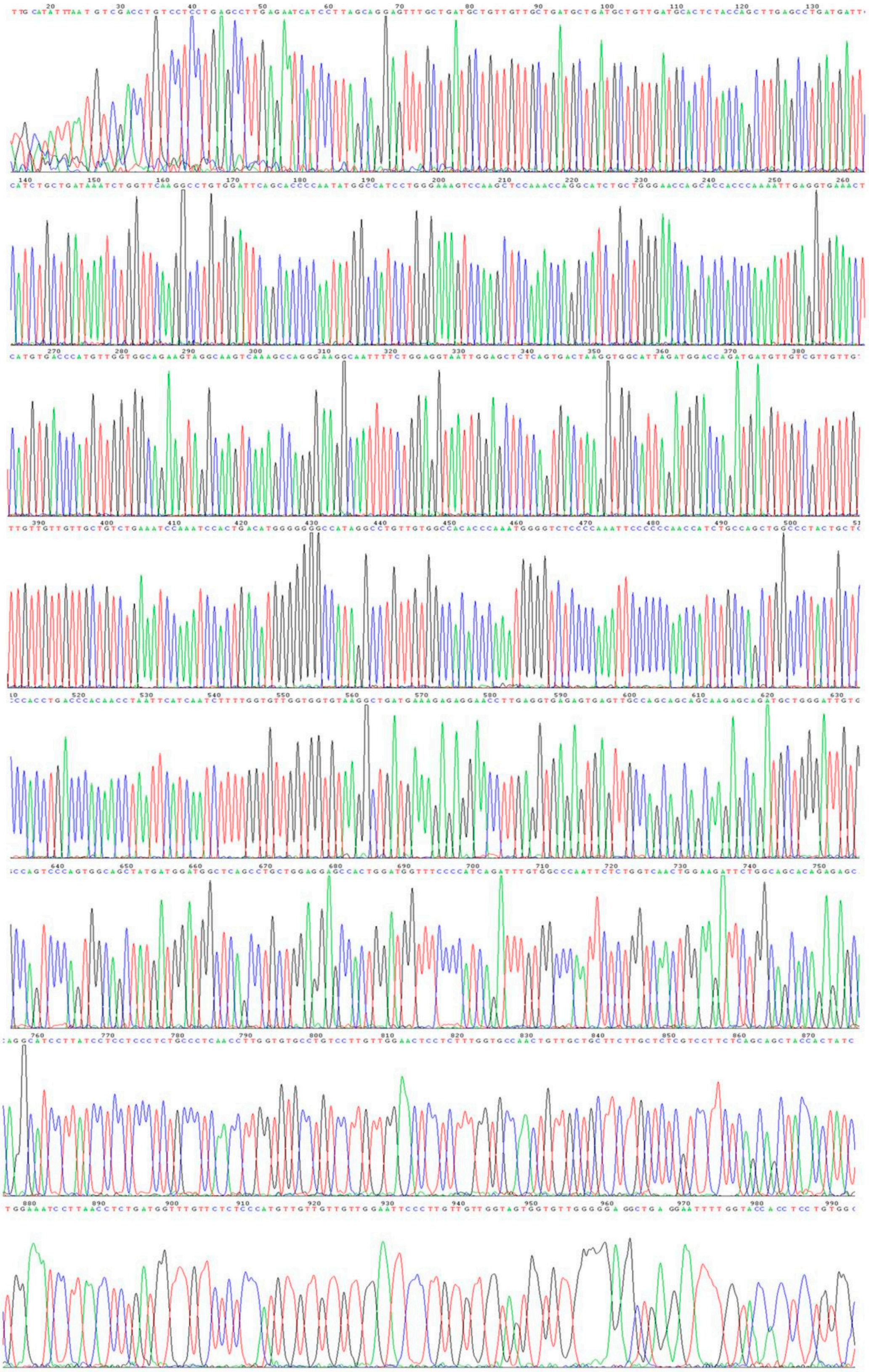

Figure S3. Sequencing of target gene from *pSOY1-Glyma.19G095300* recombinant vector for plant expression with reverse-specific primer.

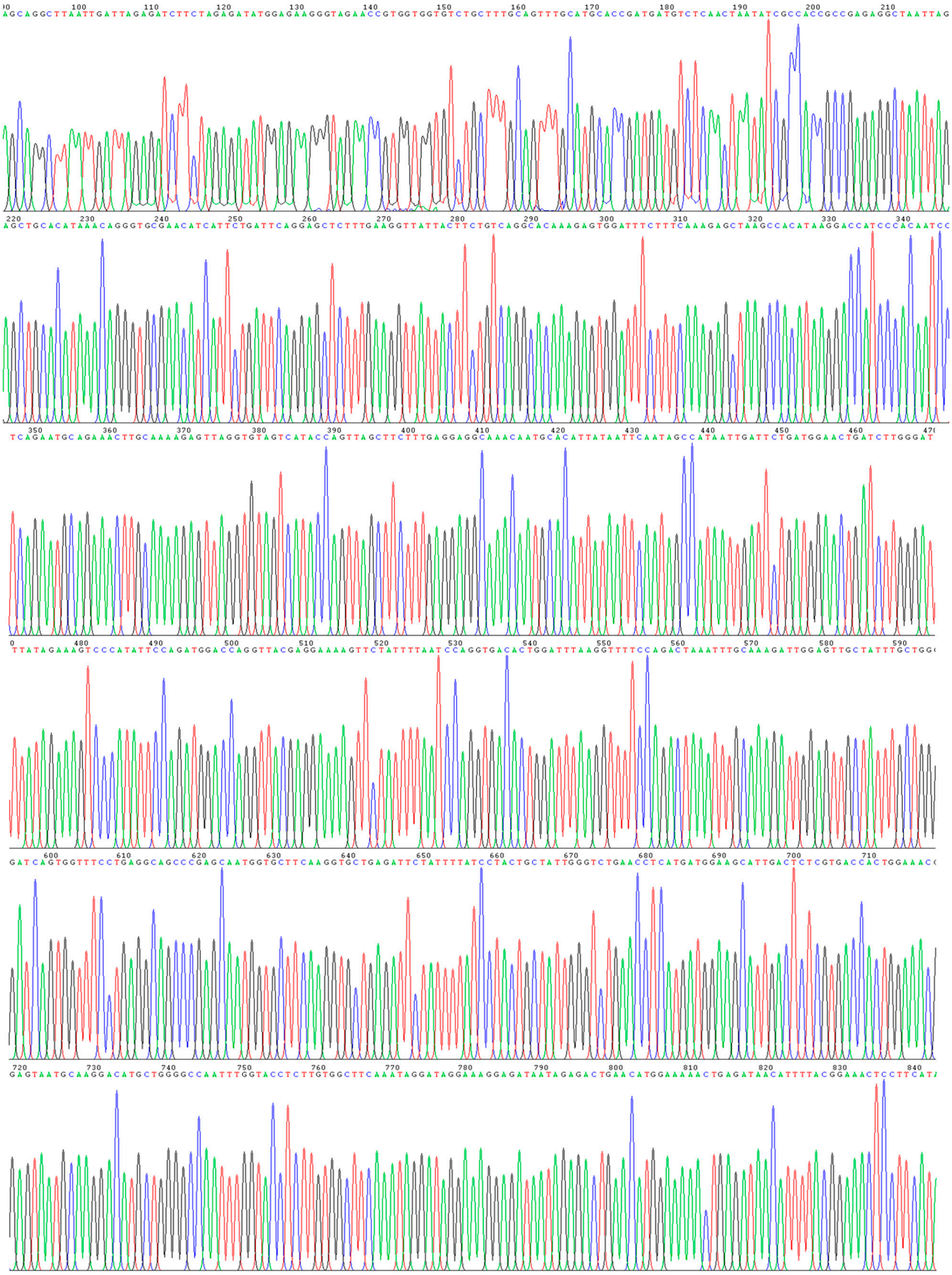

Figure S4. Sequencing of target gene from *pSOY1-Glyma.12G185700* recombinant vector for plant expression with forward-specific primer.

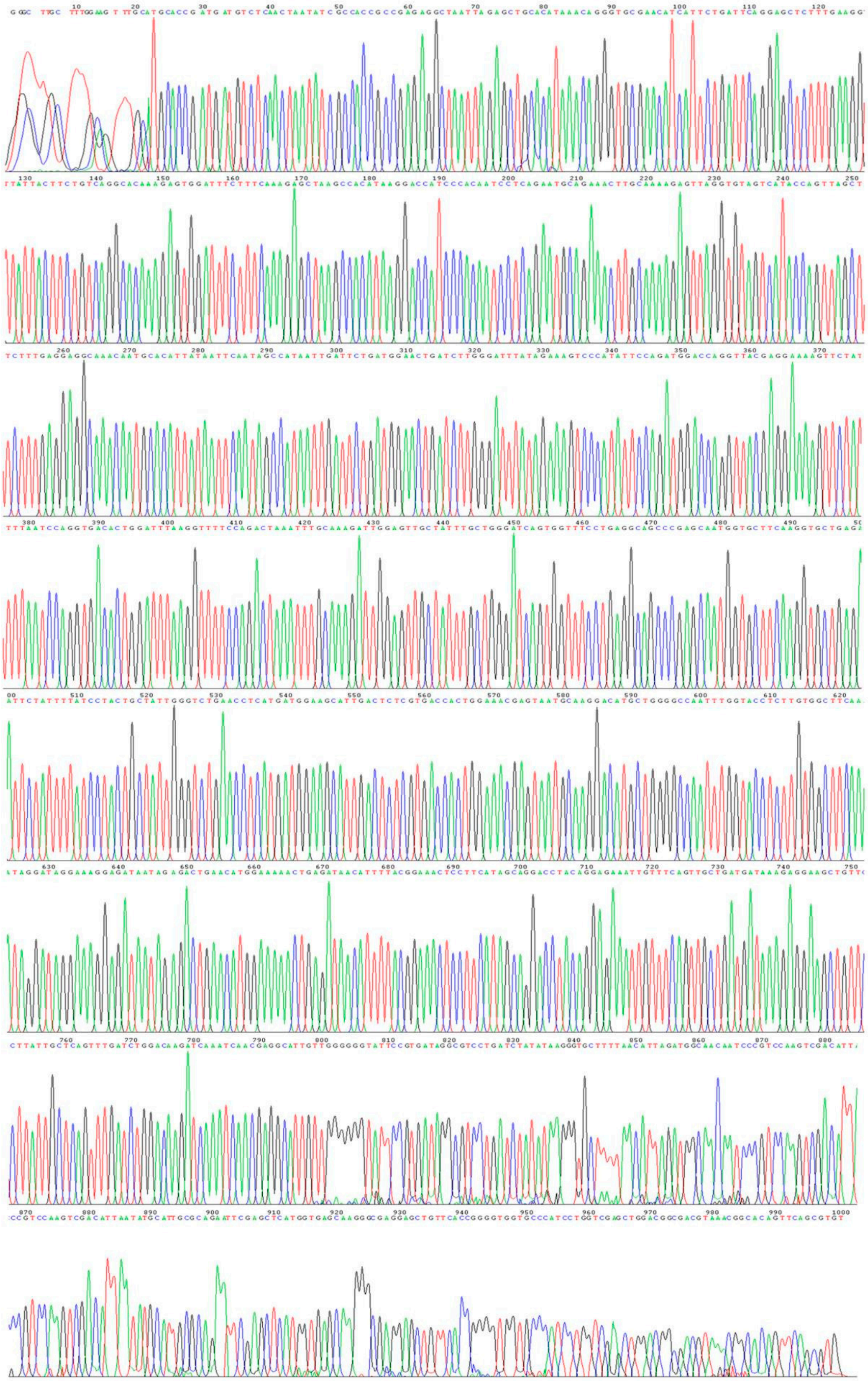

Figure S5. Sequencing of target gene from *pSOY1-Glyma.12G185700* recombinant vector for plant expression with reverse-specific primer.

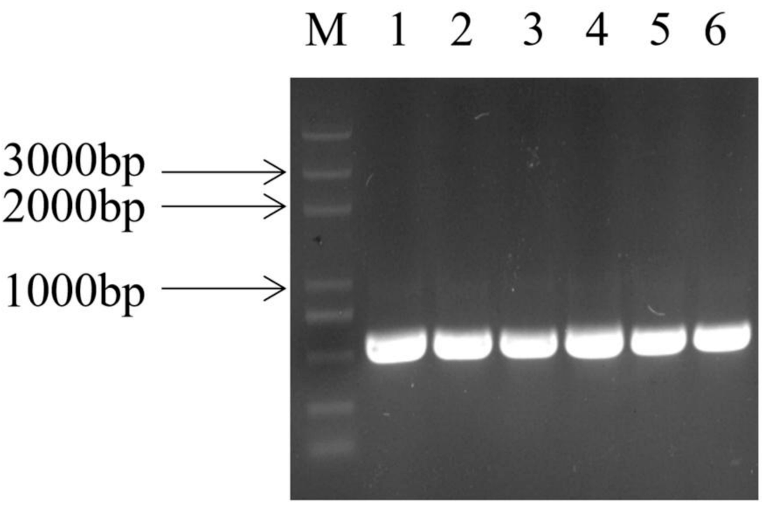

Figure S6. PCR analysis of *pGES201-Glyma.19G095300, pGES201-Glyma.12G185700* recombinant vector for CRISPR-Cas9 gene-editing.

Note: M: Trans 2K Plus DNA marker; 1, 2, 3: *Glyma.19G095300* (Crispr-F/CRISPR-TCP-R); 4, 5, 6: *Glyma.12G185700* (Crispr-F/CRISPR-NLP-R)

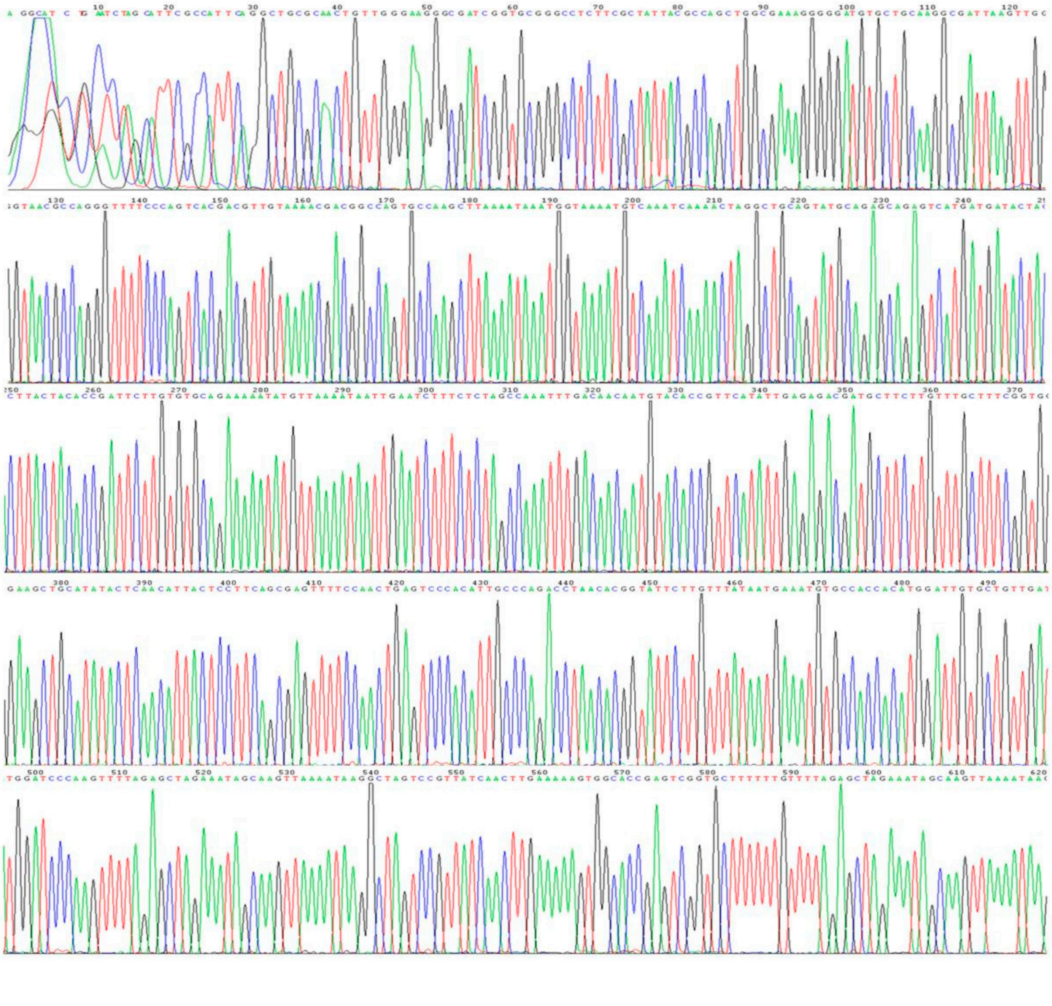

Figure S7. Sequencing of target sequence from *pGES201-Glyma.19G095300* recombinant vector.

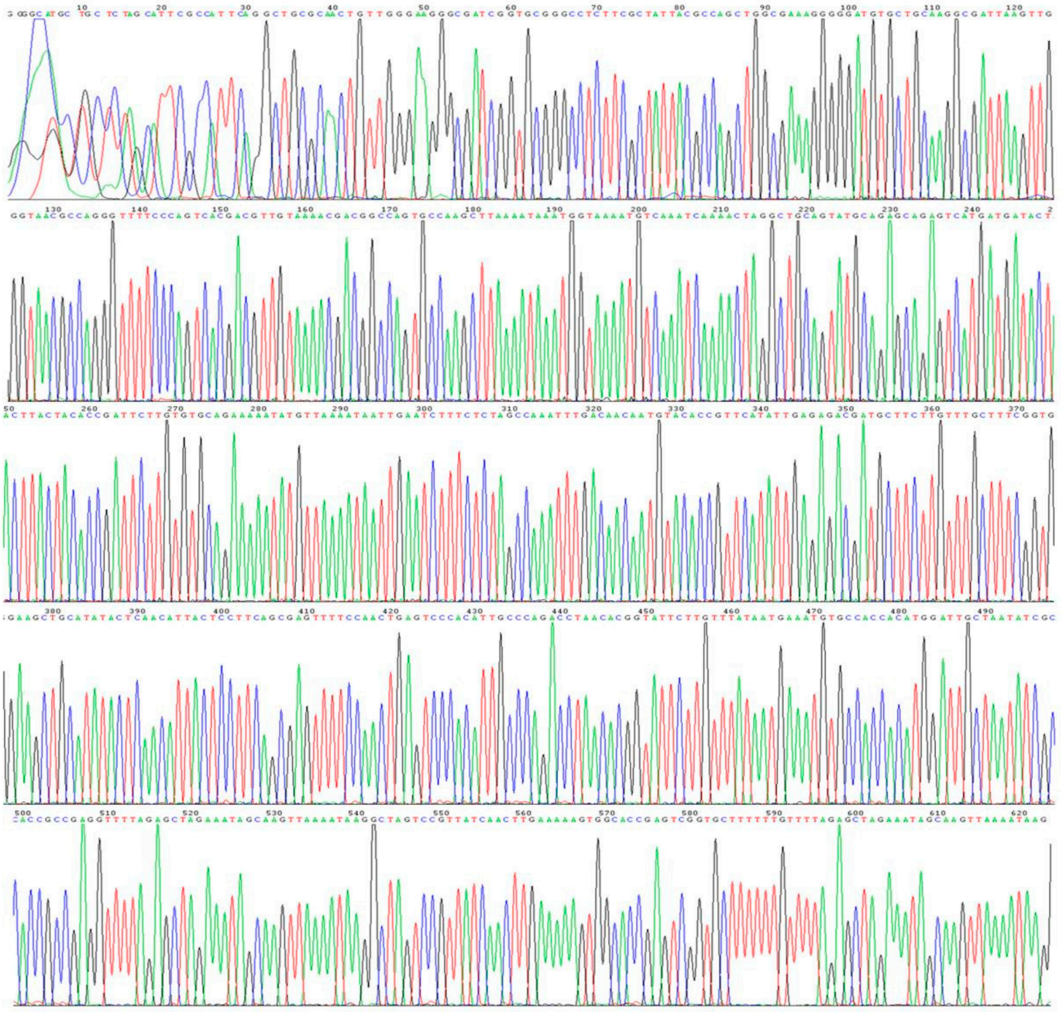

Figure S8. Sequencing of target sequence from *pGES201-Glyma.12G185700* recombinant vector.

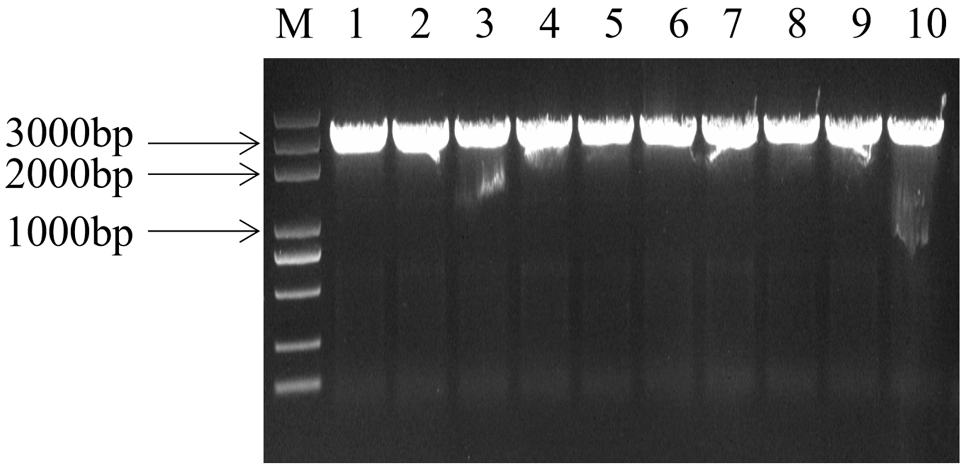

Figure S9. PCR analysis of *pCAMBIA-Glyma.19G095300pro: GUS* recombinant vector for spatial expression analysis

Note: M: Trans 2K Plus DNA marker; 1 - 10: *Glyma.19G095300pro* (pCAMBIA F/R)

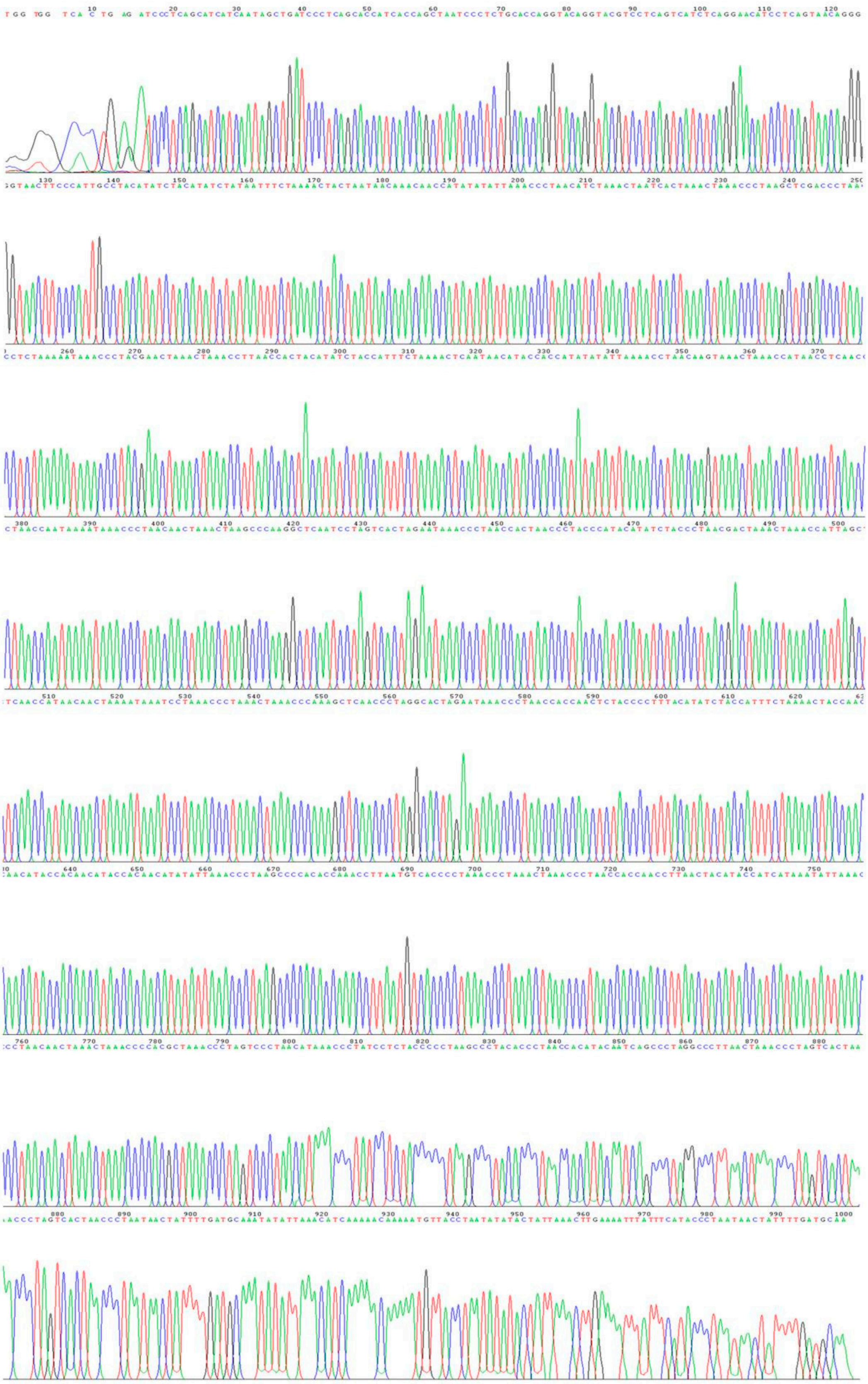

Figure S10. Sequencing of promoter sequence from *pCAMBIA-Glyma.19G095300pro: GUS* recombinant vector with forward-specific primer.
